# Exploring the Nexus between Physical and Cognitive Status in Older Adults in South Brazil

**DOI:** 10.1002/alz.088464

**Published:** 2025-01-09

**Authors:** Aline Nogueira Haas, Andréa Kruger Gonçalves, Ana Carolina Kanitz, Flávia Gomes Martinez, Leonardo Alexandre Peyré‐Tartaruga, Valéria Feijó Martins, Raphael Machado Castilhos

**Affiliations:** ^1^ Global Brain Health Institute, Dublin, Dublin Ireland; ^2^ Universidade Federal do Rio Grande do Sul, Porto Alegre, Rio Grande do Sul Brazil; ^3^ University of Pavia, Pavia, Pavia Italy; ^4^ Hospital de Clínicas de Porto Alegre, Porto Alegre, Rio Grande do Sul Brazil

## Abstract

**Background:**

Low‐middle income countries in Latin America, including Brazil, face a higher prevalence of cognitive decline compared to high‐income countries, leading to social‐economic and healthcare implications. Several studies have showed an association between reduced physical function and cognitive decline. However, there remains a gap in the understanding of this relationship within the older Brazilian population. The main objective of this study is to evaluate the physical function of older adults in South Brazil across different cognitive states.

**Method:**

A cross‐sectional analysis was conducted using baseline data from the “CREM ‐ Center for Aging and Movement Reference: a Controlled Clinical Trial” developed in the Brazilian South Region. A total of 336 community‐dwelling older adults over 60 years of age were categorized into three cognitive states based on established cutoffs points of the Montreal Cognitive Assessment (MoCA): No Cognitive Impairment (NCI), Mild Cognitive Impairment (MCI), and Cognitive Impairment (CI). Timed up and go was used to assess functional mobility; Single‐leg Stance Test to assess static balance; 10‐meter walk test to assess gait; Arm‐curl and 30‐second chair‐stand test, Back Scratch Test and Chair it‐and‐Reach Test, to assess upper and lower‐limb flexibility. The ANOVA was used to determine the main effects of the cognitive level groups (NCI, MCI and CI). A Tukey correction for multiple comparisons was used for the comparisons between groups.

**Result:**

NCI group exhibited superior functional mobility (p<0.001), balance (p<0.001), gait speed (p = 0.002), muscular endurance (p = 0.002), and flexibility (p = 0.007) compared to those with MCI and CI. All physical assessments modalities were able to differentiate the three groups: NCI was the group with better results and the MCI group showed better results than the CI group.

**Conclusion:**

This study strengthens the evidence that cognitive status influences physical function in Brazilian older adults. Identifying specific physical indicators associated with cognitive decline may enhance screening efforts in clinical trials. Longitudinal studies are recommended to elucidate the causal relationship between physical function and cognitive abilities.

